# Navigating the Labyrinth: Intensive Care Challenges for Patients with Acute-on-Chronic Liver Failure

**DOI:** 10.3390/jcm13020506

**Published:** 2024-01-16

**Authors:** Fuat H. Saner, Dimitri A. Raptis, Saad A. Alghamdi, Massimo M. Malagó, Dieter C. Broering, Dmitri Bezinover

**Affiliations:** 1 Organ Transplant Center of Excellence, King Faisal Specialized Hospital & Research Center, Riyadh 12111, Saudi Arabia; dimitri.raptis@gmail.com (D.A.R.); mdisaad@kfshrc.edu.sa (S.A.A.); crimax@me.com (M.M.M.); dbroering@kfshrc.edu.sa (D.C.B.); 2 Department of Anesthesiology and Critical Care, Hospital of the University of Pennsylvania, Philadelphia, PA 19104, USA; dmitri.bezinover@pennmedicine.upenn.edu

**Keywords:** cirrhosis, hemostasis disorder, ICU treatment, hemodynamic disorder, infection

## Abstract

Acute-on-chronic liver failure (ACLF) refers to the deterioration of liver function in individuals who already have chronic liver disease. In the setting of ACLF, liver damage leads to the failure of other organs and is associated with increased short-term mortality. Optimal medical management of patients with ACLF requires implementing complex treatment strategies, often in an intensive care unit (ICU). Failure of organs other than the liver distinguishes ACLF from other critical illnesses. Although there is growing evidence supporting the current approach to ACLF management, the mortality associated with this condition remains unacceptably high. In this review, we discuss considerations for ICU care of patients with ACLF and highlight areas for further research.

## 1. Introduction

The concept of acute-on-chronic liver failure (ACLF) was introduced by Jalan and Williams in 2002, which describes a condition with significant systemic inflammation and single or multiple organ failure (other than the liver) in the setting of acutely decompensated cirrhosis [1]. The concept was refined in 2006 by D’Amico et al. These investigators performed a systematic review of almost 120 publications examining prognostic indicators of survival in patients with compensated and decompensated cirrhosis [2]. Since then, several Chronic Liver Failure (CLIF) consortia, including Asian, European, North American, and Chinese groups, have developed different definitions and criteria for ACLF [3].

Despite some differences, all research groups agree that the main differentiating factor between decompensated liver failure and ACLF is extrahepatic organ failure [4].

ACLF and acute liver failure (ALF) have some similarities. About 50% of patients with ACLF may experience recompensation. If that is not achieved, they will require a liver transplant (LT) in the near future, similar to ALF patients.

The PREDICT study proposes to select ‘Pre-ACLF’ patients as a subpopulation of patients with cirrhosis who have an unstable clinical course and require hospitalization due to decompensation [5]. This could be sufficient for the subpopulation of patients that exhibit signs of significant inflammation or sepsis in combination with liver or/and kidney dysfunction [6]. Currently, Pre-ACLF is not a condition that is routinely identified, diagnosed, or treated.

Consequently, many patients prone to developing ACLF are not going to be identified and initially admitted to a medical floor instead of being promptly cared for in an intensive care unit (ICU) for more specialized care. Typical triggers for developing ACLF are alcohol consumption and infections, which are responsible for a significant number of acute decompensation (AD) cases associated with ACLF [3,5]. Identifying the inciting event(s) when signs of decline appear is important. Many infections leading to ACLF may not exhibit the typical signs of sepsis and can only be recognized after an AD progresses or kidney function begins to deteriorate. [5] Although infection plays a very important role in ACLF, AD, and disease progression, an infectious organism frequently cannot be identified. Infections are often hospital-acquired and may have a broad spectrum of resistance. For patients with chronic liver disease (CLD) who subsequently develop sepsis, timely administration of antibiotics is essential to prevent the development of ACLF [7]. The connection between survival rates and prompt administration of appropriate treatment emphasizes the fact that any delay in treatment has a negative effect on patient outcomes [8].

Many patients with ACLF require the administration of vasopressors to maintain a mean arterial pressure (MAP) within a range of 65–70 mmHg while avoiding fluid overload [6]. The development of grade III or IV hepatic encephalopathy (HE) indicates a poor outcome and is often linked to some type of infection [9]. Other considerations that should be addressed in this patient population include coagulation management and the prevention/treatment of acute kidney injury (AKI).

## 2. Methods

In this literature review, we critically evaluated the most current knowledge regarding pathogenesis, clinical presentation, and ACLF classification. Management of specific clinical challenges associated with this condition, as well as recently updated treatment recommendations, are included. Several databases, such as PubMed, Google Scholar, Cochrane Database, and Scopus, were analyzed using ACLF-specific keywords. Only articles published after 2005 (except one historical paper) were included. A group of experts in the field critically analyzed selected articles to formulate the recommended management strategies.

### 2.1. Definition of ACLF

Despite sharing a common pathophysiology, there are several competing definitions of ACLF proposed by different international groups, including the North American Consortium for the Study of End-Stage Liver Disease (NACSELD), the European Association for the Study of the Liver (EASL), and the Asian Pacific Association for the Study of the Liver (APASL) [10,11]. The EASL defines ACLF as hepatic decompensation complicated by other organ failures [10]. The APASL criteria include patients with or without a history of liver disease when the diagnosis of ACLF has been made [11]. Both definitions approach ACLF from different points of view. Mahmud et al., evaluated ACLF-associated mortality at 28 and 90 days using both EASL and APASL criteria [12]. Of the 80,383 patients diagnosed with cirrhosis and followed up for an average of 3.35 years, this study found that 783 patients met the criteria for ACLF according to both the EASL and APASL definitions. There were 4296 identified as having ACLF based on EASL criteria, while 574 had ACLF based on APASL criteria. The incidence of ACLF based on APASL criteria was 5.7 per 1000 person years (95% confidence interval [CI]; 5.4–6.0), whereas for EASL criteria, it was approximately 20.1 (95% CI; 19.5–20.6). Mortality rates for patients with ACLF after 28 days and 90 days according to APASL criteria were 41.9% and 56.1%, respectively, whereas these rates were slightly lower at 37.6% and 50.4%, respectively, for EASL criteria. These findings highlight discrepancies between definitions.

APASL criteria focus on liver-related factors. It includes patients with compensated cirrhosis (both diagnosed and undiagnosed) as well as those with non-cirrhotic chronic liver disease who experience their first episode of acute liver deterioration due to an acute insult to the liver, such as infection, alcohol, or trauma. The presence of jaundice (serum bilirubin ≥ 5 mg/dL) and ascites, but not serum creatinine, are standard criteria for the APASL [13].

On the contrary, NACSELD criteria do not focus exclusively on the liver. It includes patients with underlying liver disease, whether or not cirrhosis is present. NACSELD criteria require the failure of two or more organ systems beyond the liver [14]. According to the EASL CLIF (Chronic Liver Failure) criteria, ACLF is determined as a failure of one out of six organ systems. Each organ system failure is assessed using the CLIF C Organ Failure Scale (Table 1) [10].

### 2.2. System Failure and Mortality

The classification of ACLF severity based on EASL criteria is presented in Table 2 [10].

The impact of organ failure on a patient’s mortality is presented in Table 3.

### 2.3. CLIF Consortium ACLF Score

The Consortium Acute-on-Chronic Liver Failure in Cirrhosis (CANONIC) group aimed to develop a classification system capable of identifying distinct ACLF phenotypes and to establish a reliable method for predicting outcomes in this patient population [17]. The CANONIC study group introduced grades of ACLF from 1 to 3. A modified SOFA score was used to assess each organ system, creating a linear severity scale of up to 100. This grading system found that one-month mortality was 20% to 70% if scores were between 45 and 64. However, when the CLIF-C-ACLF score was above 64, the mortality rate approached 90%. This score can be evaluated daily and can be used as a useful tool to monitor response to treatment. This score, which incorporates the CLIF-C Organ Failure Score, age, and white blood cell count, acts as a dynamic tool for prognosis. For further details, please see Table 4.

The calculation can be found at the following website: https://www.efclif.com/scientific-activity/score-calculators/clif-c-aclf. (accessed on 1 September 2023).

### 2.4. Cirrhosis-Associated Immune Dysfunction

After a precipitating event, cirrhotic patients develop a systemic inflammatory response. This response is associated with the upregulation of cytokines and the release of damage-associated molecular patterns (DAMPs), with subsequent activation of Toll-like receptors [18]. The pathophysiological features seen in patients with ACLF are distinct from the response seen after acute decompensation of cirrhosis without organ failure [4]. The excessive systemic inflammatory response can lead to significant circulatory instability with impaired oxygen delivery. This can further worsen tissue perfusion, ultimately leading to multi-organ failure [19].

Bacterial infections and alcohol consumption frequently occur as initiating events for ACLF. Bacterial infections activate the immune system through pathogen-associated molecular patterns (PAMPs), which stimulate the production of pro-inflammatory cytokines and mediators, leading to hepatocellular injury. This cellular injury, in turn, results in the release of DAMPs, which exacerbate the inflammatory response. Consequently, hepatocytes compromised by inflammation and/or necrosis add to the inflammatory environment, generating metabolites that can further propagate systemic organ dysfunction and immune dysregulation [4].

Alcohol directly compromises immune responses and facilitates the translocation of bacteria in the intestine, with subsequent escalation of inflammation and disruption of normal immune function. Additionally, immunosuppressive therapies, which are sometimes employed in the management of alcoholic hepatitis, might also play a role in the development of ACLF. These therapies can enhance the existing condition of immune paresis, which in turn may increase susceptibility to bacterial infections [20].

**Figure 1:**
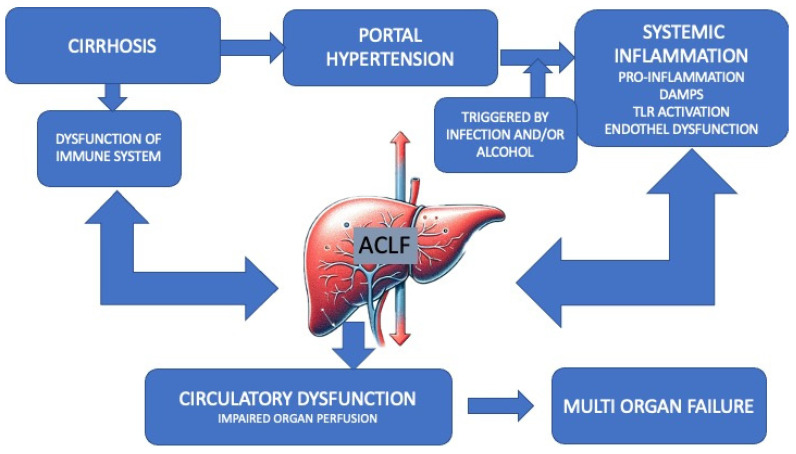
Pathogenesis of ACLF.

There is a growing body of evidence suggesting that patients with cirrhosis may undergo changes in their intestinal microbiota, which can result in impairment of their intestinal barrier defense mechanisms. These changes are often proportional to the severity of liver disease (Figure 1) [21].

The figure represents the multifactorial etiology and progression from cirrhosis to acute-on-chronic liver failure (ACLF). In patients with pre-existing liver cirrhosis, certain precipitating incidents can trigger ACLF via multiple mechanisms, such as amplification of immune dysregulation and activation of pathogenic processes, including endothelial dysfunction and the production of proinflammatory cytokines. These processes synergistically contribute to the progression toward organ failure and the onset of ACLF modified according to Zaccherini [14]. ACLF, acute-on-chronic liver failure. DAMP: damage-associated molecular pattern. TLR4, toll-like receptor 4.

### 2.5. Admission of Patients with ACLF to the ICU

An increasing number of patients with acute-on-chronic liver failure (ACLF) are being admitted to the ICU. Research indicates an improvement in ICU survival rates for these patients [22]. Unfortunately, ICU mortality rates are still high, particularly within certain subgroups. Patients admitted to the ICU due to gastrointestinal hemorrhage tend to have a better prognosis compared to those with septic shock. Additionally, early admission to the ICU is associated with a favorable outcome. These factors should be carefully considered when making decisions about ICU admissions for patients with cirrhosis.

Outcomes for critically ill patients with cirrhosis in the ICU are dependent on the presence and severity of other organ failure, which can be assessed using various scoring systems. The CLIF-Sequential Organ Failure Assessment and the CLIF-C in patients with ACLF have been shown to have better prognostic value compared to general ICU scores such as the Acute Physiology And Chronic Health Evaluation (APACHE) II, the Simplified Acute Physiology Score (SAPS) II, or liver-specific scores such as the Model for End-Stage Liver Disease (MELD) or Child Turcotte Pugh [23].

### 2.6. The Essentials of Cardiovascular Care

Chronic liver disease is always associated with systemic inflammation and endothelial dysfunction. As a result, cirrhotic patients develop severe vasoplegia, which leads to stagnation of blood flow, particularly in the portal system [24,25]. Vasoplegia in patients with cirrhosis is related to the release of nitric oxide, which leads to an increased cardiac output (due to a lower systemic vascular resistance) and hypotension in combination with a decrease in systemic blood volume [3,26]. Furthermore, individuals with this condition may also develop a related end-stage liver disease-specific cardiomyopathy and/or portopulmonary hypertension, which can further affect the cardio-pulmonary system. Several events, including infection, alcohol consumption, trauma, bleeding, and surgical procedures, can lead to cardiovascular system failure [6]. The main goal of volume resuscitation is to ensure efficient tissue oxygen delivery by maintaining adequate perfusion pressure. Studies on patients with cirrhosis associated with septic shock have demonstrated that having a MAP higher than 70 mmHg provides no benefit [3]. A recent evaluation in septic patients did not find any difference in mortality between the high-pressure target group (aiming for MAP 80–85 mmHg) and the low-pressure target group (aiming for MAP 65–70 mmHg) at 28 and 90 days. Adverse events were similar in both groups, but the high-pressure target group had a slightly higher rate of new-onset atrial fibrillation. It was demonstrated that patients in the high-pressure target group with known hypertension more frequently required renal replacement therapy (RRT), although this did not affect mortality.

Data regarding patient management demonstrated that these patients were most frequently cared for in the ICU. Fluid overload is a significant problem in this patient population due to increased portal pressure. Assessment of fluid responsiveness is recommended before beginning resuscitation, and crystalloids have been demonstrated to be the preferred fluid [27]. It has also been shown that the administration of balanced electrolyte solutions is preferable in comparison to 0.9% saline. Normal saline causes an imbalance in chloride levels, which can lead to acidosis, hyperkalemia, and a higher risk of developing AKI [28,29]. Using starch-based solutions for resuscitation is also not recommended due to an increased risk of developing AKI [30,31,32]. Based on current knowledge, it is still being determined if the administration of albumin is beneficial [33], although some studies indicate that if hemodynamic stability is not achieved following the administration of crystalloids, albumin may be helpful to achieve better hemodynamics [34,35]. Albumin administration is logical, considering that albumin is important in medication binding, exhibits antioxidant properties, and helps regulate both the immune system and endothelial activity [36]. However, studies in patients with cirrhosis fail to demonstrate any survival benefit for albumin administration, with the exception being in patients with spontaneous bacterial peritonitis in the setting of sepsis [37,38].

If tissue perfusion is inadequate or blood pressure remains low despite fluid administration, it is advisable to use vasopressors. There is evidence from studies performed in patients with septic shock having hemodynamic characteristics similar to those seen in patients with ACLF that strongly supports norepinephrine as the preferred vasopressor [39]. Epinephrine administration is often associated with the deterioration of tissue perfusion and subsequent increases in lactate production [40]. However, in a randomized controlled trial (RCT) investigating patients with sepsis, no significant distinction was found between the administration of epinephrine and the combination of norepinephrine and dobutamine [41]. At day 28, the epinephrine group had a 40% mortality rate, while the norepinephrine plus dobutamine mortality rate was 34% (*p* = 0.31). Mortality rates upon ICU discharge, hospital discharge, and by day 90 were similar between the two groups. Additionally, the time to achieve stability after discontinuation of vasopressors and the progression of SOFA scores were comparable. Both groups demonstrated similar rates of adverse events. Despite the side effects associated with vasopressin and its derivatives, the Surviving Sepsis Guidelines recommend using vasopressin as an additional agent for managing septic shock after other options have been exhausted. This recommendation is based on clinical studies and meta-analyses that indicate a reduction in catecholamine use and a noticeable increase in blood pressure if vasopressin is added to the treatment protocol [35]. Currently, there is no evidence suggesting that this approach can significantly improve survival.

### 2.7. The Use of β-Blockers in Patients with Sepsis or ACLF Is Still a Topic of Discussion

Non-selective beta blockers (NSBBs) are frequently prescribed for patients with cirrhosis to manage portal hypertension and prevent variceal bleeding. The CANONIC study, which included 349 patients, demonstrated that patients with ACLF admitted to the hospital and managed with NSBBs had improved survival at 28 days. This improvement may be attributed to reduced bacterial translocation in the intestine, decreasing systemic inflammation [42]. There is debate regarding the cardiac protection of β-blockers [43]. A recent meta-analysis performed by Tan et al., suggested that administering β-blockers before the development of sepsis might potentially reduce mortality rates [44]. Based on current knowledge, there are no definitive reasons to stop the use of NSBBs [45].

### 2.8. Acute Kidney Injury: Intervention and Care

AKI often develops as a complication of ACLF, impacting about half of patients with CLD admitted to the hospital. It serves as an indicator of decreases in both long- and short-term survival [46].

Hepatorenal syndrome (HRS) is frequently associated with ACLF and historically has been divided into two subtypes: HRS Type 1 and Type 2. HRS Type 1 usually appears suddenly within a two-week period and is associated with a poor prognosis. HRS Type 2 usually has a prolonged course and is associated with moderate kidney dysfunction and ascites that do not respond well to diuretics [47]. The definition of HRS has been updated to align with the criteria for AKI. The International Club of Ascites adjusted the terminology and diagnostic criteria for HRS Type 1, redefining it as HRS-AKI. This change was performed after investigations indicated that elevated serum creatinine levels at treatment onset correlated with a decreased likelihood of reversible HRS [48]. In patients with ACLF who are at risk of developing AKI, it is important to ensure adequate renal blood flow with fluid administration (and inotropes as necessary) to maintain a MAP between 65 and 70 mmHg along with an appropriate cardiac index. The administration of vasopressors should be prioritized in situations of volume overload [49]. In these situations, the use of terlipressin as an infusion is generally better tolerated and potentially more effective compared to administering this medication as a bolus. It has been demonstrated that if AKI treatment is initiated early, there is an increased likelihood that the kidney will recover, which is associated with improved survival [50]. A comprehensive analysis suggests that in terms of reversing HRS and reducing mortality within 30 days, the effectiveness of norepinephrine is comparable to terlipressin [51].

The timing of initiating dialysis is important for patient outcomes. Over the years, there have been five RCTs attempting to determine when to initiate RRT for patients with AKI II and III. Among these studies, the three largest and most comprehensive (AKIKI, IDEAL-ICU, and STARRT AKI) demonstrated that starting RRT early did not result in improved survival. Additionally, it was demonstrated that there were risks associated with early RRT, including RRT dependency, an increased likelihood of bacteremia and catheter-related complications, bleeding problems, and higher resource consumption [52,53,54]. However, a smaller, single-center study (ELAIN) in cardiac surgical patients demonstrated significantly improved survival if RRT was started early [55] (Table 1). According to current data, the delayed strategy seems justified unless there are life-threatening indications.

The “Artificial Kidney Initiation in Kidney Injury-2” (AKIKI-2) study evaluated the impact of two different waiting strategies (length of time) for starting RRT in 278 patients [56].

In contrast to the original AKIKI study’s late arm, AKI was defined by oligo-anuria > 72 h and/or a urea-N concentration > 112 mg/dL. Only then were patients randomized 1:1 into a “late” strategy where RRT was started < 12 h after randomization or into an “even-later” strategy when RRT was started only under absolute emergency indications (hyperkalemia > 6 mmol/L, metabolic acidosis pH < 7.15, pulmonary edema) or when serum urea-N exceeded 140 mg/dl. Oligo-anuria was not a trigger for starting RRT, even in the “even-later” arm. The primary endpoint of this study was “days alive and without RRT” within the first 28 days, if this persisted for more than three days. There was no difference regarding the number of days without RRT. There were also no differences in secondary endpoints such as “ventilation-free days”, “duration of ICU stay”, or “recovery of kidney function”.

In the IDEAL-ICU study, 17% of patients in the late group had to undergo emergency dialysis and experienced a higher mortality rate. These findings raise the question of the appropriate time to start RRT in patients with AKI III without emergency indications.

In the AKIKI, IDEAL-ICU, and STARRT-AKI trials, an early start was compared to waiting up to 72 h. If urgent indications do not develop after 72 h, a late start of RRT can still be dangerous, as was demonstrated in the AKIKI-2 study. In the “even-later” arm of this study, 79% of patients actually had to undergo dialysis. This was very similar to the ELAIN study, where over 90% of patients had to be dialyzed after waiting. In both studies, waiting was associated with a worse outcome (Table 5).

These studies have clearly demonstrated that patients who inevitably need dialysis do not benefit from waiting because of the high rate of complications. However, an individualized approach should be used: patients likely to recover are not subjected to dialysis, but for those likely to require dialysis, it should be started as soon as practical. This is especially true for patients with ACLF who are already fluid-overloaded. Classic dialysis indicators such as high serum potassium may not be accurate due to an increase in the extracellular space.

Patients with ACLF often demonstrate hypercoagulability. Under these circumstances, anticoagulation for any kind of extracorporeal therapy, particularly RRT, is necessary. While laboratory results might indicate a predisposition for bleeding, there is frequent filter clotting in the absence of anticoagulation. Many intensive care specialists, however, are hesitant to employ regional citrate anticoagulation for these patients due to concerns about citrate toxicity. More recently, numerous studies have demonstrated the safety and effectiveness of using regional citrate anticoagulation for RRT in patients with cirrhosis who required RRT while on the waiting list and in newly transplanted patients [57,58].

### 2.9. Infections in Patients with ACLF

The management of infectious conditions in patients with ACLF is very complex. Around 46% of individuals admitted to the hospital due to worsening cirrhosis are diagnosed with bacterial infections. In two-thirds of these cases, the infections are identified only upon admission. Recent data indicates that SBP are particularly common in this patient population, accounting for around 20–30% of infections. Urinary tract infections are responsible for up to 20–25%, pneumonia 20%, bloodstream-related infections 8–15%, and infections affecting the skin and soft tissues between 5 and 10% [59,60].

Prompt initiation of antibacterial agents when bacterial etiology infection is presumed can markedly enhance survival. An investigation including over 600 patients with septic shock demonstrated that postponing antibacterial therapy post-onset of hypotension was associated with increased mortality. This study also demonstrated that the use of inappropriate antibiotics led to an increased risk of mortality (adjusted odds ratio 9.5, 95% CI: 4.5–20.7) [8]. During the last twenty years, the spectrum of bacterial infections among patients with cirrhosis has significantly changed. The use of quinolone antibiotics unexpectedly led to an increase in the prevalence of gram-positive infections. Frequent prescriptions of third-generation cephalosporins have unintentionally contributed to a rise in enterococci infections, as these bacteria naturally resist cephalosporins. This trend is particularly prominent with nosocomial infections. This concerning development is associated with the increasing occurrence of bacterial resistance due to multiple treatment regimens using antibiotics that are available as opposed to the most appropriate. This evolution demonstrates the need for appropriate antibiotic management and innovative treatment approaches [59,60].

Fernández et al., conducted a study to investigate patterns of resistance and the presence of multidrug-resistant organisms (MDRO) [61]. Their research revealed a significantly increased incidence of MDRO in patients with ACLF, from 29% to 38% between 2011 and 2017. Bacterial pathogens found included those that produce extended-spectrum beta-lactamase, particularly within the Enterobacteriaceae family. Key factors independently linked to the spread of MDR infections (MDRI) include the presence of nosocomial infections, admission to the ICU, and recent hospital admissions [61]. To control the spread of MDRI in patients with cirrhosis, stringent preventative and prompt treatment measures are crucial. Empirical treatment strategies using broad-spectrum MDR antibiotics have been shown to be superior to traditional approaches, particularly in cases of nosocomial infections and severe sepsis [61]. The use of beta-blockers to treat portal hypertension in patients with ACLF has been shown to be effective in attenuating inflammation, decreasing the grade of ACLF, and improving outcomes [42].

Managing infections effectively in the setting of generally increasing antibiotic resistance requires customization of medication choice and treatment durations. It is also important to transition from broad-spectrum antibiotics to organism- and sensitivity-specific treatments as soon as possible.

Patients with cirrhosis often exhibit fungal colonization when hospitalized, but the prevalence of systemic infection remains under 5%. Aspergillosis is a specific concern for patients with cirrhosis. Aspergillosis can significantly compromise the course of the disease and even be fatal. Furthermore, patients with cirrhosis and aspergillosis can also develop candidemia as well as further complications related to cytomegalovirus infection [62]. Regular infection surveillance is essential for success.

Patients with ACLF in the ICU have a high risk of fungal infections [63]. Alexopoulou et al., performed an analysis of 185 hospitalized patients with cirrhosis with confirmed infections.

The authors demonstrated that the overall incidence of fungal infections was 10%, while 4.3% of patients had both fungal and bacterial infections, and 6% had only fungal infections [64].

A recent meta-analysis evaluated contributing factors for fungal infections (FIs) in patients with cirrhosis [65]. The authors analyzed 34 studies involving a total of 2134 patients. The analysis revealed that patients with FIs had a mortality rate 2.1 times higher compared to those without FIs. Patients with FIs also had higher Child Turcotte Pugh and MELD scores, multiple organ failures, and an increased hospital length of stay.

The highest mortality (76.6%) was observed in patients with ACLF and FIs. Patients with aspergillosis of the lung had a mortality rate of 79.4%, peritoneal FIs of 68.3%, and bloodstream FIs of 55% [65]. It seems reasonable to begin antifungal and antibiotic treatment for patients with ACLF as soon as they are admitted to the ICU. The urgency to treat must be carefully weighed against the risks associated with the overuse of these agents, particularly broad-spectrum antibiotics, which are effective against a wide range of bacteria, making them invaluable when the specific cause of an infection is unknown. Their overuse, however, is significantly related to developing antimicrobial resistance (AMR). To address this concern, the medical community has embraced the concept of antimicrobial stewardship, which involves a multi-discipline team responsible for selecting appropriate antibiotic therapy based on culture and sensitivity data and adjusting treatment as soon as more specific information becomes available. These initiatives aim to optimize antibiotic use to ensure effective treatment while at the same time reducing the risk of AMR [66,67].

### 2.10. Biomarkers

Early detection of sepsis is crucial for the successful treatment of ACLF. In the past 30 years, many biomarkers have undergone an evaluation regarding their reliability for the early diagnosis of ongoing infections. One study examined procalcitonin (PCT) and C-reactive protein (CRP) [68]. Patients in this study were categorized as either infected or non-infected and assessed using median levels of PCT and CRP. Levels of PCT in the non-infected group were found to be 0.4 ng/mL, while those with localized infections had levels at 1.4 ng/mL (*p* < 0.001). Median levels of CRP were 79.9 mg/L for patients without an infection and slightly higher at 85.3 mg/L for those with localized infections (*p* < 0.001). The area under the curve (AUC) for PCT is 0.756, compared to 0.580 for CRP.

In cases of systemic inflammation, the median values of PCT rose significantly to 3.65 ng/mL compared to 0.4 ng/mL in non-septic patients. Similar results were shown for CRP: 115.6 mg/L in septic patients compared to 79.9 mg/L in patients without sepsis. The AUC for PCT was 0.925 and 0.677 for CRP. Another group found different results regarding the predictive value of PCT in patients after LT [69]. Of the 135 LT recipients, 22% had serious infections. Those with infections had significantly higher PCT and CRP levels, as well as a slightly increased white blood count. This study found that CRP, but not PCT, was an independent risk indicator for predicting infections. This suggests that CRP could be useful in guiding treatment after LT.

There are also some limitations to the use of these biomarkers. Relatively low sensitivity and even lower specificity are significant limitations of CRP. Serum concentrations of this biomarker can be found in burn injury and malignancy and in patients with significant cardiovascular disease [70,71]. It has also been demonstrated that there is no correlation between CRP level and the severity of sepsis [72]. Similar limitations were shown for PCT. PCT was found to be increased in critically ill patients with both microbiologically proven and not-proven sepsis [73]. A number of other potentially promising sepsis biomarkers are under evaluation, including a subtype of the CD14 receptor presepsin, a soluble form of triggering receptors expressed on myeloid cells, sTREM-1, CD64, interleukin-6, and TNF-α. Currently, there is no evidence that any particular biomarker should be prioritized for predicting the development of sepsis, assessing sepsis severity, or evaluating the effectiveness of sepsis therapy.

### 2.11. How the Brain Responds: Cerebral Reactions

Hepatic encephalopathy (HE) is frequently associated with cirrhosis and is primarily related to decreased metabolization of ammonia. Patients with HE usually experience confusion and decreased mental status but can have seizures and even progress to coma. In patients with ACLF, HE is significantly associated with increased mortality. Fortunately, significant cerebral swelling is very rare. It has been demonstrated that the prevalence of cerebral edema is about 4%, but if it occurs, it is associated with a worse outcome [74]. The etiology of HE is very complex, and its presentation depends on several factors. One of these is an increased concentration of serum ammonia, which is primarily produced by intestinal bacteria and not metabolized in the cirrhotic liver. Other factors contributing to the development of HE include hyponatremia, damage to neurons with disruptions in the blood-brain barrier, and abnormalities in the GABAergic and benzodiazepine pathways with subsequent impaired neurotransmission [75]. In patients with ACLF, decreased mental status is not just related to hyperammonemia but also to generalized inflammation that affects cranial blood vessels, endothelium, and astrocytes [76]. Ongoing infections can worsen existing HE even more. If a patient’s conscious level decreases (HE ≥ III°), airway protection with endotracheal intubation may become necessary. Factors contributing to the development of HE, including infection, constipation, dehydration, and electrolyte abnormalities, should be investigated and managed. All medications that can alter a patient’s mental status should be discontinued if possible. Treatment of hyperammonemia with lactulose or non-absorbable antibiotics such as rifaximin (which minimizes nitrogen uptake from the intestines) is frequently started, but their efficacy for patients with ACLF remains uncertain [77]. The HELP study compared polyethylene glycol 3350 electrolyte solution (PEG) and the usual lactulose treatment for HE [78]. The findings indicated that PEG might be more effective. Within a 24 h period, 91% of patients in the PEG group had significant improvement vs. 52% in the standard therapy group. An RCT compared the effects of L-Ornithine L-Aspartate (LOLA) on patients diagnosed with HE. One hundred forty patients were assigned in a 1:1 ratio (70 patients in each group) to receive LOLA in combination with lactulose and rifaximin vs. placebo in combination with lactulose and rifaximin as a control group. LOLA was administered for five days. The primary endpoint was an improvement in HE. This study demonstrated that the LOLA cohort had faster recovery from HE and a significantly lower mortality rate (16.4% vs. 41.8%) than the control group [79].

A limitation of the protein intake (a hyperammonemia management option frequently applied in the past) proved not to be helpful and even could be associated with deterioration of the patient’s nutritional status. If medication therapy fails, RRT should be performed [80].

There are other novel methods under investigation to reduce ammonia levels. One of them is the use of ST-120 produced by the Kureha Corporation in Tokyo, Japan. ST-120 is a spherical carbon adsorbent taken orally. It is approved in Japan for its role in postponing the need for dialysis and alleviating symptoms of uremia in patients with advanced chronic kidney disease (CKD) [81]. It lowers indoxyl sulfate (IS) levels in the bloodstream, a uremic toxin known to accelerate CKD progression. AST-120 suppresses the gastrointestinal absorption of indole, a byproduct of tryptophan breakdown and a precursor to IS [82].

The Cochrane Database evaluated five medications used to control ammonia levels for individuals with cirrhosis to prevent and treat hepatic encephalopathy. This study focused on randomized clinical trials (RCTs) [83]. Eleven RCTs met the criteria for evaluation. Included trials compared study medications to a placebo or non-disaccharide. This analysis includes 3 evaluations of sodium benzoate effectiveness, 1 evaluation of glycerol phenylbutyrate, 2 with ornithine phenylacetate, 2 with AST 120, and 3 additional evaluations with polyethylene glycol. The main findings from the review were that sodium benzoate, glycerol phenylacetate, and AST 120 were effective in reducing blood ammonia levels compared to placebo. However, compared to absorbable disaccharides, these medications did not show a significant difference regarding their ability to lower serum ammonia levels. Both glycerol phenylbutyrate and polyethylene glycol have demonstrated potential in the effective management of encephalopathy compared to placebo or lactulose. Importantly, none of the evaluated medications had an impact on the risk of death.

Liver assist devices are sometimes used to treat HE [84]. Although this has not been demonstrated to improve the mortality of patients with cirrhosis, a significant reduction in ammonia levels has been demonstrated in several studies [85,86]. None of these studies, however, were able to demonstrate any survival benefit [87,88].

### 2.12. The Dynamics of Coagulation in Patients with ACLF

Patients with ACLF are prone to both bleeding and clotting complications [89]. Patients with cirrhosis and/or ACLF have a rebalanced hemostasis affecting all branches of the coagulation system. While these patients frequently have thrombocytopenia, other factors may offset the bleeding risks. The concentration of both procoagulants and anticoagulants (proteins C, S, and antithrombin III) is all decreased, while the concentration of liver-independent coagulation factors (F), including FVIII and von Willebrand factor (vWF), is 3–4 times higher in patients with ACLF compared to healthy controls. The concentration of ADAMTS13 (produced in the liver and responsible for vWF degradation) is markedly decreased, which limits the bleeding risk but increases the risk of clotting [90,91]. It has been demonstrated that in the population of hospitalized patients with CLD who have been acutely decompensated, thrombin generation (TG) is significantly increased, which significantly increases the risk of thromboses. This is even more pronounced in patients with ACLF [92]. However, significant fibrinolysis driven by sepsis-related organ failure in patients with ACLF might balance this prothrombotic effect [93].

Standard laboratory tests (SLT) are not designed to predict bleeding or thrombosis. They can only evaluate serum levels of procoagulants and do not reflect global hemostasis [94]. Although SLTs are still commonly employed for coagulation monitoring, these tests are not accurate in predicting spontaneous bleeding risks or procedure-induced bleeding [95,96]. Viscoelastic tests (VETs), such as thromboelastography (TEG) or Rotational Thromboelastometry (ROTEM), evaluate whole blood samples and assess the interaction between both pro- and anticoagulants and platelets. Indeed, two randomized controlled trials found that using VETs to guide transfusion in patients with ACLF demonstrated a reduction in the use of blood products without an increase in spontaneous or procedure-related bleeding [97,98]. Administering fresh frozen plasma (FFP) to patients with cirrhosis without signs of bleeding is generally not recommended [99] and can lead to an unnecessary increase in circulating volume and increases in portal pressure, with a subsequent increased risk of bleeding [100]. It has also been demonstrated that FFP administration has a minimal impact on TG [99]. Instead of FFP, the administration of specific coagulation factors can now be considered. The most frequently used compounds are fibrinogen concentrate, prothrombin complex concentrate (PCC) (encompassing factors II, VII, IX, and X, proteins C and S, heparin, and antithrombin III), and factor XIII [101]. It has been demonstrated that the use of these factor concentrates leads to significantly increased TG in patients with cirrhosis, which theoretically might increase the thrombotic risk [102]. In clinical settings, however, the use of these medications has been demonstrated to be safe. It has been shown that if VETs were used to guide the administration of factor concentrates, the incidence of thrombosis would not increase [103]. PCC, however, should not be administered to patients without bleeding signs and VET monitoring [104].

Thrombocytopenia occurs frequently in patients with sepsis. There is a strong correlation between thrombocytopenia and multiorgan failure (MOF), which is often associated with a poor outcome [105]. The pathophysiology of thrombocytopenia-associated MOF is related to reduced function of the enzyme ADAMTS13, which leads to decreased breakdown of vWF multimers. This results in the buildup of extremely large von Willebrand factor multimers. These oversized vWF multimers facilitate platelet clumping and intravascular clotting, causing hemolysis (the formation of schistocytes), ultimately leading to ischemia and failure of various organs [106]. VETs cannot detect thrombocytopenia or dual antiplatelet inhibition. However, ROTEM can identify impairments in the platelet contribution to clot formation. By comparing the maximum clot firmness (MCF) of EXTEM and FIBTEM tests, it is possible to discern whether the problem is due to a deficiency in fibrinogen or inadequate platelet aggregation [89].

Integrating VETs into coagulation management involves a comprehensive approach that includes understanding the test, correct interpretation of results, and implementing treatment. Developing clear standard protocols and guidelines is critical to integrating VET test results into patient care plans.

### 2.13. Beyond Conventional Treatments: The Impact of Liver Assist Devices

#### 2.13.1. Bioartifical Liver Assist Device

The Extracorporeal Liver Assist Device (ELAD^®^, developed by Vital Therapies Inc., San Diego, CA, USA) utilizes hepatocytes derived from a hepatoblastoma cell line. A phase III clinical trial involving 203 participants with severe alcoholic hepatitis (AH) was recently performed [107]. The trial protocol randomized participants to receive either a combination of 3–5 days of continuous ELAD therapy and standard care (SOC) or just the SOC. An intent-to-treat analysis demonstrated there was no difference in patient survival between groups (51.0% for ELAD + SOC vs. 49.5% for SOC alone). A subgroup analysis of patients with a MELD score below 28 and an age under 46.9 years found a better (but statistically not significant) 90-day survival rate in the ELAD group compared to the SOC group (100% vs. 73%, *p* = 0.08). A subsequent evaluation targeting AH patients with more specific patient demographics (n = 151) found no significant survival benefit: the 90-day mortality rate was 19.2% for ELAD+SOC and 21.9% for SOC alone (*p* = 0.68). (available only as an abstract) [108].

#### 2.13.2. Non-Biological Devices

The Prometheus^®^ system (Fresenius Medical Care AG, Hamburg, Germany) is using a Fractionated Plasma Separation and Adsorption (FPSA) approach. This system combines plasma separation with adsorption techniques, facilitating the removal of both albumin-bound and water-soluble toxins. In the HELIOS study, 145 patients with ACLF demonstrated reduced serum bilirubin levels in this study group in comparison to controls. There was, however, no survival benefit [88].

The Molecular Adsorbent Recirculating System (MARS) (Baxter International Inc., Deerfield, IL, USA) device operates using an external blood circuit with a semi-permeable, albumin-coated membrane. The part of the system is a dialysis circuit containing 600 mL of 20% human albumin with a charcoal column and an anion exchange resin column (to remove albumin-bound toxins) with a conventional hemodialysis filter. The RCT (RELIEF trial) compared MARS treatment in patients with ACLF with SOC [87]. A total of 189 patients with hypoalbuminemia, HE II-IV, and/or HRS were recruited for this study. Each patient in the study group received 6.5 ± 3.1 MARS sessions. There was no difference regarding transplant-free survival after 28 and 90 days.

These trials, however, did not utilize the ACLF criteria proposed by the CANONIC study. In a recent meta-analysis, data from 285 participants from three RCTs involving MARS treatment in patients with CLD were reassessed [109]. According to the updated ACLF criteria, 165 cases were reclassified as ACLF. In both groups (ACLF and non-ACLF), MARS was able to significantly decrease bilirubin levels and improve HE but failed to demonstrate any difference in 90-day survival.

### 2.14. Futility Criteria for Liver Transplantation

Futility in organ transplantation is a topic of ongoing discussion. When considering the limited resources and very high cost associated with LT in patients who are frequently extremely deconditioned, finding a fair and balanced approach to achieve the best outcome is often very difficult [110].

Various authors have attempted to propose distinct definitions of futility in transplantation. Rana et al., identified transplantation as futile if mortality after LT is higher than the mortality on the waiting list. [111] Petrowski et al., developed a futility risk model using four factors, including patient MELD score and comorbidities, cardiac risk, and the presence of septic shock preoperatively [112]. Panchal et al., focused on the 90-day mortality rate as a measure of futility [113]. There is no unified definition for futility used to determine when to refuse LT, which poses a significant challenge in daily clinical practice. Making the decision-making process more objective would be an important step toward establishing clear limits. To achieve this goal, a survey across 35 transplant centers in the United States was conducted [114]. A significant majority of clinicians favored proceeding with LT for candidates with high MELD scores, even if they required a higher level of support in the ICU. Criteria to refuse LT included a high dose of vasopressors to maintain hemodynamics, a fraction of inspired oxygen of 70% and above to maintain oxygenation, and sepsis associated with gram-negative rods. Additionally, 56% of transplant clinicians considered severe frailty (non-ambulatory or wheelchair-bound) as not compatible with LT.

In a recent consensus document, a multidisciplinary group of experts proposed that severe frailty associated with failing organs and ongoing sepsis, especially if not adequately treated with antimicrobials within 72 h, would be a reason to postpone LT [115]. The consensus on thresholds for severe organ failure contradicting LT included a PaO_2_/FiO_2_ ratio of less than 150 mmHg, a norepinephrine dose exceeding 1 µg/kg per minute, or a serum lactate level greater than nine mmol/L.

Due to the lack of clear criteria to deny LT, decisions currently need to be made on a case-by-case basis. Implementation of delisting criteria in allocation systems for candidates deemed too ill for LT could make the decision-making process more objective. Such a system would relieve some pressure on treating physicians and allow for a more thorough evaluation of outcomes.

Numerous clinical studies have been conducted over the last three decades in an attempt to demonstrate the benefits of liver assist devices. Many of these evaluations have shown partial benefits, such as the successful treatment of hepatorenal syndrome, or HE. More than 20 years ago, there was significant enthusiasm that these extracorporeal procedures might offer a similar life extension seen with hemodialysis in patients with end-stage-renal failure. Except for one study on acute liver failure [116] (carried out with plasmapheresis), however, a clear survival advantage remains to be demonstrated. Using assist devices as a bridge to transplant in some selected critically ill patients with ACLF seems justifiable, but they cannot generally be recommended as a standard treatment.

## 3. Conclusions

The concept of ACLF has been around for over a decade, and numerous publications have emerged since then that have helped refine both the definition and criteria related to the condition as well as inform patient management. The care of this patient population remains extremely challenging. Management of infections and coagulopathy, HE, AKI, and cardiovascular instability frequently require early ICU admission. Prompt administration of appropriate antibiotic therapy and early dialysis for patients with AKI grade II and above is essential. Coagulation management should be guided by VET, and the preemptive use of blood products should be avoided. Liver support devices show promise to serve as a bridge to LT until an appropriate hepatic graft becomes available.

## Figures and Tables

**Table 1:** Differences between EASL/APASL and NACSELD regarding definition of ACLF (modified according to [14]).

Criteria	EASL	APASL	NACSELD
Definition	Acute worsening of preexisting liver disease, often triggered by an event, leads to increased 4-week mortality due to organ failure.	Acute deterioration of the liver leading to jaundice and hemostasis disorder within 4 weeks causes ascites/encephalopathy in known or unknown chronic liver disease with high 4-week mortality.	Chronic liver disease, with or without cirrhosis, causes mortality within 3 months if left untreated.
Liver Failure Criteria	Bilirubin > 12 mg/dL	Bilirubin > 5 mg/dL, INR > 1.5, or prothrombin activity < 40%	Not specified
Extrahepatic Failure	Renal: Creatinine ≥ 2.0 mg/dL or dialysis; Brain: West Haven HE grades 3–4; Circulation: any Vasopressor use; Respiration: PaO_2_/FiO_2_ ≤ 200 mmHg, SpO_2_/FiO_2_ ≤ 214, or mechanical ventilation	Renal: Dialysis; Brain: West Haven HE grades 3–4; Circulation: Shock (MAP < 60 mm Hg); Respiration: Mechanical ventilation	Renal: Dialysis; Brain: West Haven HE grades 3–4; Circulation: Shock presence (MAP < 60 mmHg); Respiration: Mechanical ventilation is required
Type of Acute Insult	Primarily, alcohol and bacterial infections	Primary viral infections	Primarily bacterial infections, not specified
Timeframe of Acute Insult	Not specified	Within 4 weeks	Within 3 months
Disease Severity Assessment	CLIF-SOFA score	No specific score	No specific score

ACLF: acute-on-chronic liver failure; APASL, Asian Pacific Association for the Study of the Liver. CLIF-SOFA: Chronic Liver Failure-Sequential Organ Failure Assessment. EASL: European Association for the Study of the Liver. HE: hepatic encephalopathy. INR: international normalized ratio. NACSELD: North American Consortium for the Study of End-Stage Liver Disease.

**Table 2:** Grading of acute-on-chronic liver failure (ACLF) as per the European Association for the Study of the Liver (EASL) (according to [15]).

ACLF Grade	Clinical Presentation
No ACLF	No organ failure or single non-kidney organ failure; creatinine <1.5 mg/dL; no hepatic encephalopathy (HE)
ACLF Grade 1	Single renal failure OR single non-kidney organ failure, creatinine 1.5–1.9 mg/dL, and/or HE grades 1–2
ACLF Grade 2	Two organ failures
ACLF Grade 3	Three or more organ failures

**Table 3:** Mortality rates for each grade of ACLF at different time intervals (adapted from [16]).

ACLF Classification	Mortality Day 28	Mortality Day 90	Mortality Day 180
No ACLF	10%	24%	38%
ACLF Grade 1	21%	42%	47%
ACLF Grade 2	57%	74%	79%
ACLF Grade 3	87%	95%	96%

**Table 4:** Chronic Liver Failure Consortium Organ Failure (CLIF-C OF) Score. Adapted according to Jalan et al. [17].

Organ/System	Variable	Score = 1	Score = 2	Score = 3
Liver	Bilirubin (mg/dL)	Less than 6	6 to less than 12	12 or more
Coagulation	INR	Less than 2	2 to less than 2.5	2.5 or more
Kidney	Creatinine (mg/dL)	Less than 2	2 to less than 3.5	3.5 or more, or renal replacement therapy
Brain	Encephalopathy grade (West Haven Criteria)	Grade 0	Grade 1–2	Grade 3–4
Circulation	MAP (mm Hg)	70 or more	Less than 70	Use of Vasopressors
Respiratory	PaO_2_/FiO_2_ ratio	More than 300	More than 200 and 300 or less	200 or less
	SpO_2_/FiO_2_ ratio	More than 357	More than 214 and 357 or less	214 or less

**Table 5:** Comparison of 4 RCTs.

	ELAIN [55] Zarbock et al.	AKIKI [53] Gaudry et.al	IDEAL-ICU [52] Barbar et. al	START-AKI [56] Investigators Group
Patients (n)	231	620	477	2927
Setting	95% (Cardiac surgery)	80% Sepsis	100% septic Shock	67% medical patients
Criteria early	AKI II° and NGAL ≥ 150 ng/mL	AKI III	Stage F of RIFLE	AKI II
Criteria late	AKI III	Urgent indicaton for dialys3s	48 h Remaining at Stage F	Urgent indication
Primary endpoint	90 day mortality	60 day mortality	90-day Mortality	90-day mortality
Mortality early group	39% (*p* = 0.03)	49%	58%	44%
Mortality Late group	55%	50%	54%	44%
Mortality Dialyzed Late Group	NA	62%	68%	NA
Rate of non-dialysis in late group	9.2%	49%	38%	38%
Dependence on dialysis on 90-days after randomization	13 vs. 15	2 vs. 5	2 vs. 3	10.4. vs. 6; RR 1.74 95% CI [1.24–2.43]

## Data Availability

Not applicable.
